# Supplementary material for: Targeted Anticancer Agent with Original Mode of Action Prepared by Supramolecular Assembly of Antibody Oligonucleotide Conjugates and Cationic Nanoparticles
Source: Pharmaceutics. 2023 Jun 2;15(6):1643. doi: 10.3390/pharmaceutics15061643 (PMC10305667; doi:10.3390/pharmaceutics15061643)
Supplement: Supplementary file 1 [file pharmaceutics-15-01643-s001.zip › pharmaceutics-2329707-supplementary.pdf]

Targeted anticancer agent with original mode of action prepared by supramolecular assembly of antibody oligonucleotide conjugates and cationic nanoparticles.

Supplementary information

Conjugates' characterization:

Antibody-oligonucleotide conjugates DoC distribution determination by SDS PAGE

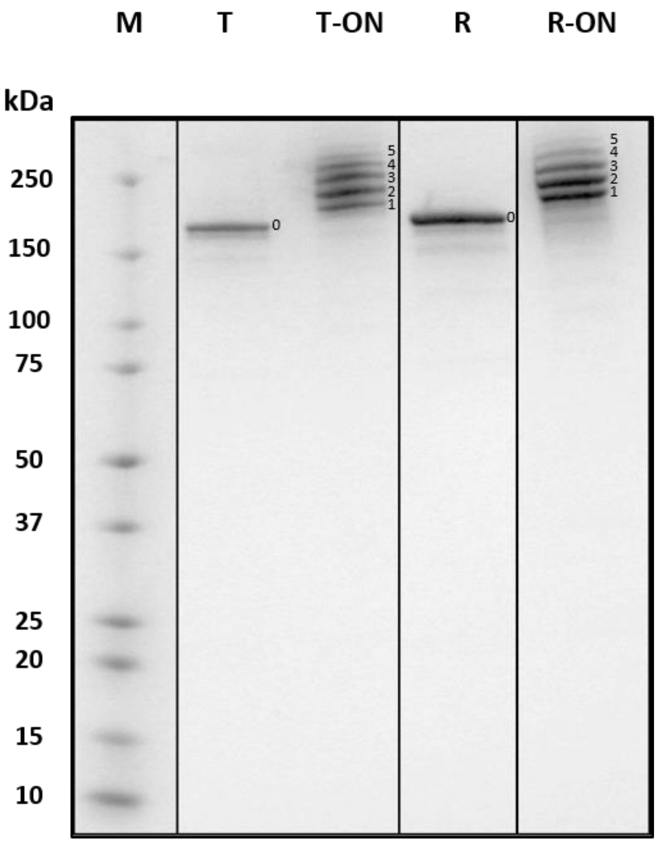

Figure S1: DoC distribution observed by SDS-PAGE (4-15%). Next to each lane the DoC of the corresponding conjugate is indicated by a number. T, trastuzumab; R, rituximab; ON, oligonucleotide (ssDNA). Mean $DoC_{(T\text{-}ON)}$ = 2.9; mean $DoC_{(R\text{-}ON)}$ = 2.9.

Native-MS analysis of azido-modified antibodies

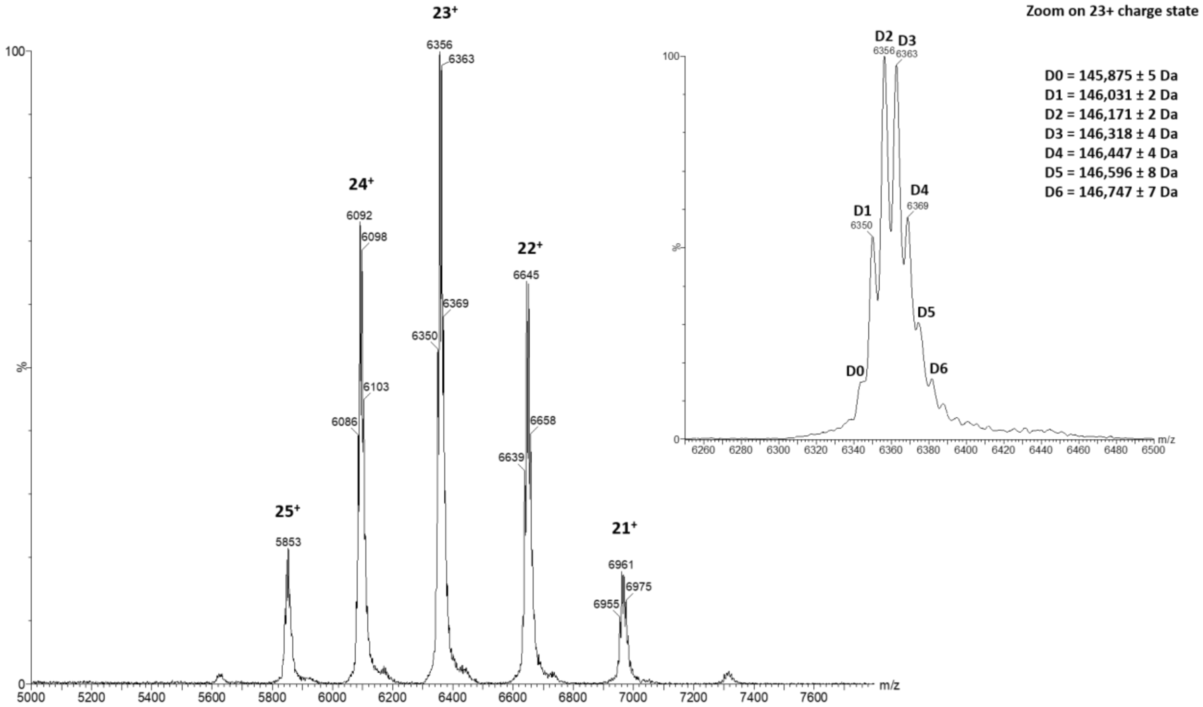

Figure S2: Mass spectrum (left) and zoom on 23+ charge state (top right) of the azido-modified Trastuzumab intermediate. Mean DoC = 2.8

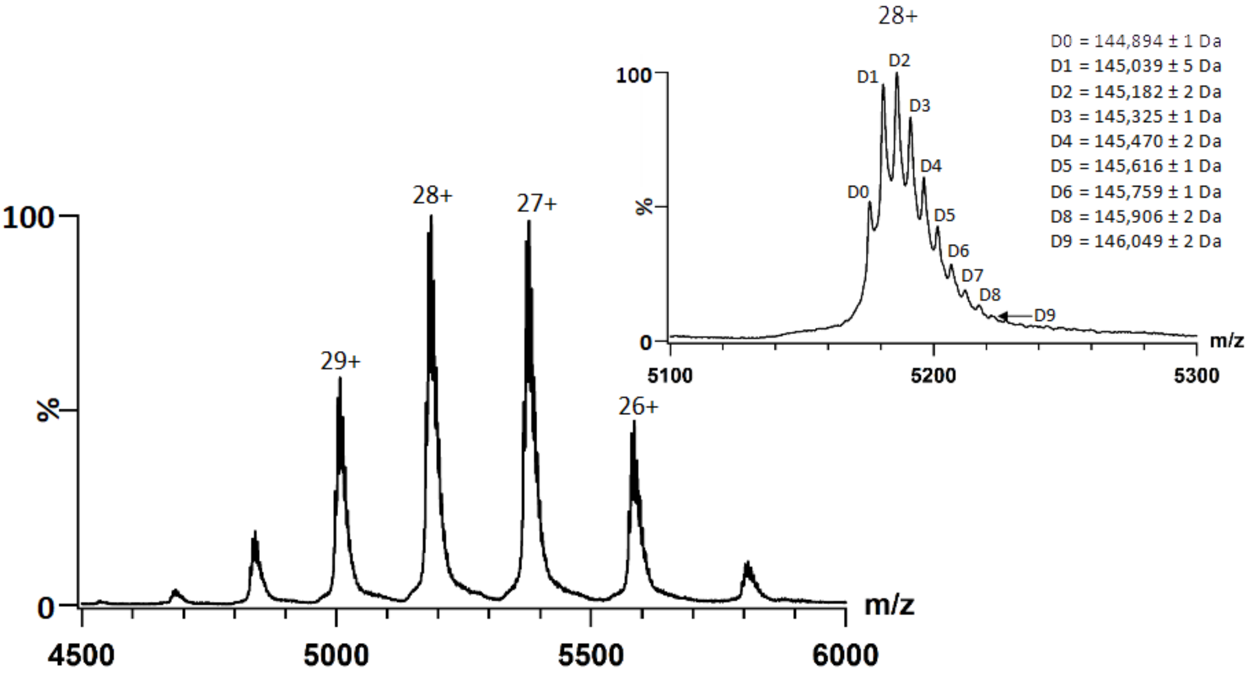

Figure S3: Mass spectrum (left) and zoom on 28+ charge state (top right) of the azido-modified Rituximab intermediate. Mean DoC = 2.8

Trastuzumab-ssDNA conjugate Degree of Labelling (DoL) determination

After FITC-labelling, the fluorescein concentration of the T-ssDNA conjugate was measured by absorption spectrophotometry using NanoDrop's "proteins and labels" mode.
The DoL was calculated using eq. S1.

$$DoL = \frac{fluorescein\ concentration\ (M)}{Protein\ concentration\ (M)} \quad (eq.\ S1)$$

The antibody concentration was determined by BCA assay (see above).

Complex characterization

Gel mobility shift assay

Table S1: formulation of complexes made for gel mobility shift assay in Fig S4.

		**Gel mobility shift assay with dye-labeled T-ssDNA conjugate**						
**T-ssDNA/Micelle ratio**		**0.1**	**0.2**	**0.4**	**0.7**	**1.0**	**2.0**	**-**
**Volumes drawn from stock* (µL)**	**cNP**	6.93	3.47	1.73	0.99	0.69	0.35	0
	**T-ssDNA**	2						
	**HBG**	3.07	6.53	8.27	9.01	9.31	9.65	10
**Concentrations in the final ATNP solution (µM)**	**cNP**	2.63	1.31	0.66	0.38	0.27	0.13	-
	**T-ssDNA**	0.26						

* Stock solutions concentrations: T-ssDNA 0.23 g/L (i.e. 1.58 µM) in PBS, Micelle 4.55 µM of particle (i.e. 1mM of monomer) in water.

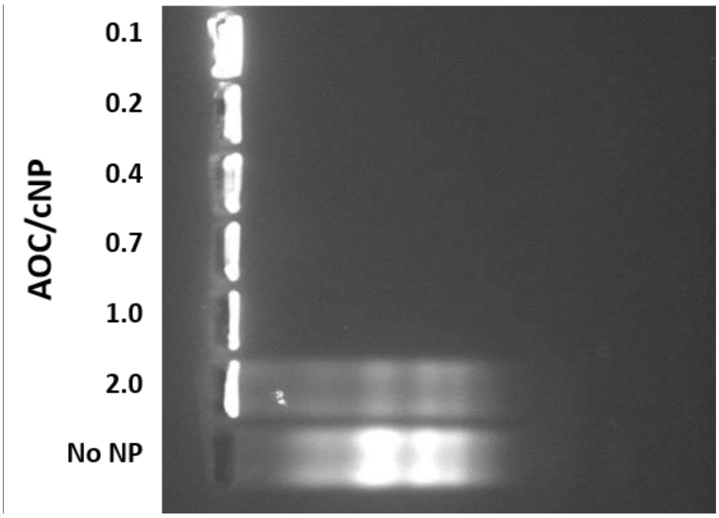

Figure S4: Gel mobility shift assay corresponding to Table S1. To identical aliquots of T-ssDNA AOC were added various quantities of cationic micelles, forming complexes of indicated AOC/cNP ratios. The cationic micelles are able to complex AOC at up to mean AOC/cNP ratio = 1.0

Table S2 formulation of complexes made for gel mobility shift assay in Fig S5.:

		Gel mobility shift assay with dye-labeled ssDNA							
**ssDNA/Micelle ratio**		**0.3**	**0.4**	**0.6**	**1.2**	**2**	**3**	**5.9**	**-**
**Volumes drawn from stock** (μL)**	**cNP**	9.17	6.87	4.58	2.29	1.37	0.92	0.47	0
	**ssDNA**	1							
	**HBG**	1.83	4.13	6.42	8.71	9.63	10.08	10.53	11
**Concentrations in the final ATNP solution (μM)**	**cNP**	3.47	2.60	1.74	0.87	0.52	0.35	0.18	-
	**ssDNA**	1.04							

** Stock solutions concentrations: ssDNA 12.5 μM in PBS, Micelle 4.55 μM of particle (i.e. 1mM of monomer) in water.

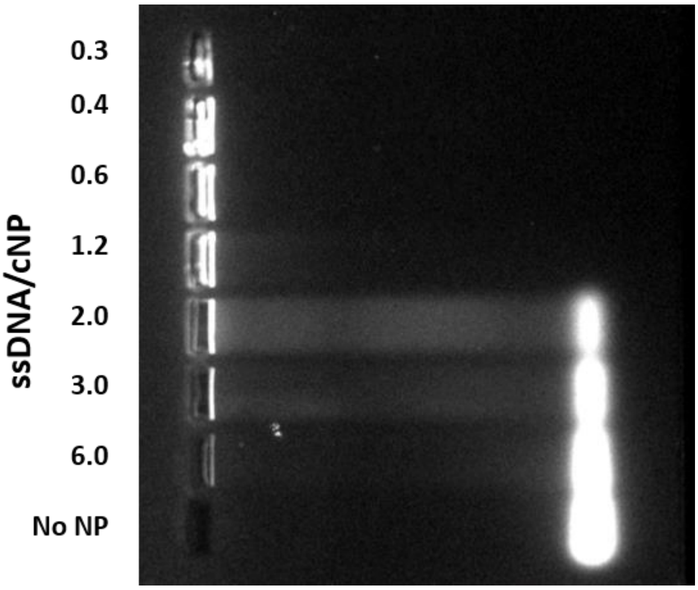

Figure S5: Gel mobility shift assay corresponding to Table S2. To identical aliquots of ssDNA AOC were added various quantities of cationic micelles, forming complexes of indicated ssNA/cNP ratios. The cationic micelles are able to complex ssDNA at up to mean AOC/cNP ratio = 1.2

Size and ζ potential measurements

Table S3: For each formulation, this charts provides the size and ζ values obtained ± standard deviation of three runs

AOC/cNP	Size (nm)	ζ potential (mV)
**0**	7.3 ± 1.5	+ 76.3 ± 8.5
**0.1**	60.0 ± 13.3	+ 18.6 ± 7.0
**0.2**	39.7 ± 8.3	+ 10.1 ± 0.0
**0.4**	73.0 ± 13.7	+ 4.1 ± 0.0
**0.7**	229.5 ± 38.5	- 0.0 ± 0.0
**1.0**	204.6 ± 4.0	- 6.5 ± 0.0

Table S4: Formulation of complexes for cytotoxicity assays, size and ζ potential measurements.

		Cytotoxicity *in vitro*, size & zeta assays						*In vivo* (volumes for one injection)		
**AOC/Micelle ratio**		**0**	**0.1**	**0.2**	**0.4**	**0.7**	**1.0**	**0.4 (ATNP)**	**cNP control**	**Vehicle control**
**Volumes drawn from stock*** (μL)**	**cNP**	220						9.9	9.9	0
	**AOC**	0	64	127	254	444	635	11.4	0	0
	**HBG**	780	716	653	526	336	145	23.7	35.1	45
**Concentrations in the final ATNP solution (μM)**	**CcNP**	1						1		0
	**C_{AOC}**	0	0.1	0.2	0.4	0.7	1	0.4	0	

*** Stock solutions concentrations: AOCs (i.e. T-ssDNA and R-ssDNA): 0.23 g/L (i.e. 1.58 μM) in PBS, Micelle 4,55 μM of particle (i.e. 1mM of monomer) in water.

In vitro experiments

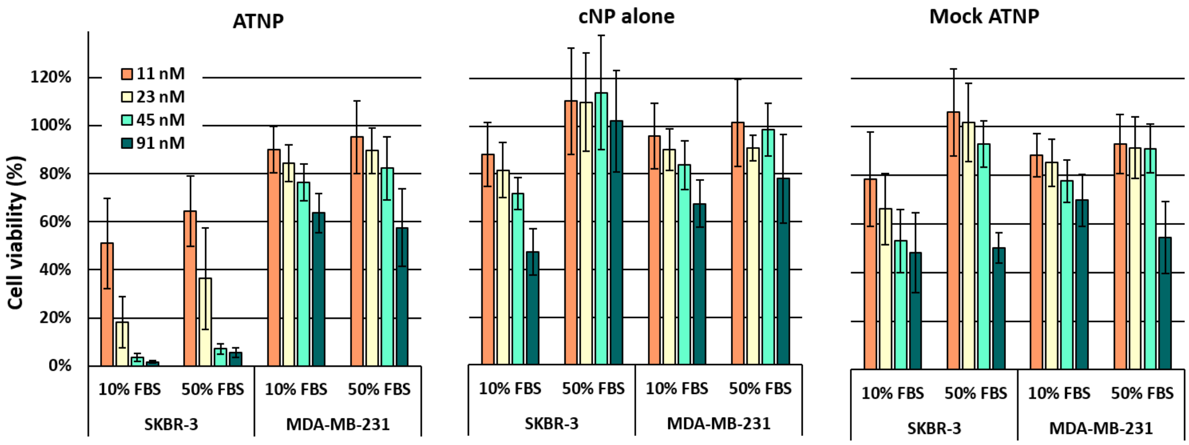

Figure S6: The cellular viability of both the HER2$^+$ SKBR-3 and the HER2$^-$ MDA-MB-231 cell lines after 96 h of continuous incubation at 37 °C in either 10% or 50% FBS supplemented culture medium, and in the presence of various particles concentrations of either the ATNP (T-ssDNA/micelle Antibody-Toxic Nanoparticles complex), the mock ATNP (R-ssDNA/micelle complex) or the cNP (cationic micelle), was measured using the MTT assay. For the ATNP and mock ATNP, the complexes were prepared at a molar ratio of 0.4 AOC/cNP. The viability is expressed as a percentage, relative to vehicle-treated (i.e. 20 µL of pure HBG buffer), and the error bars represent the standard deviation calculated from at least 3 independent assays.

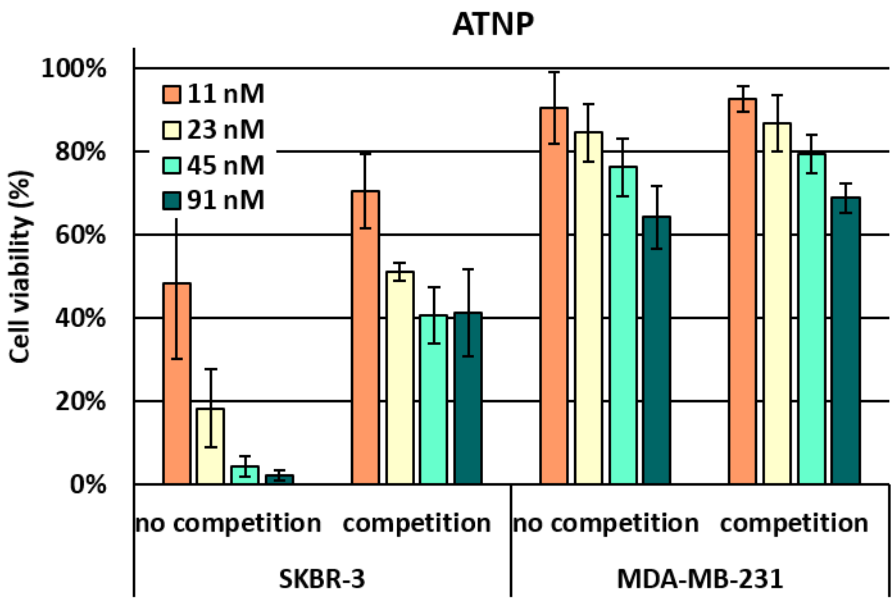

Figure S7: The cellular viability of both the HER2$^+$ SKBR-3 and the HER2$^-$ MDA-MB-231 cell lines after 96 h of continuous incubation at 37 °C in culture medium, in the presence of either 0 (no competition) or 1 µM of free anti-HER2 antibody trastuzumab (competition), along with various particles concentrations of the ATNP (T-ssDNA/micelle Antibody-Toxic Nanoparticle complex), was measured using the MTT assay. The ATNP complex were prepared at a molar ratio of 0.4 AOC/cNP. The viability is expressed as a percentage, relative to vehicle-treated (i.e. 20 µL of pure HBG buffer), and the error bars represent the standard deviation calculated from at least 3 independent assays.

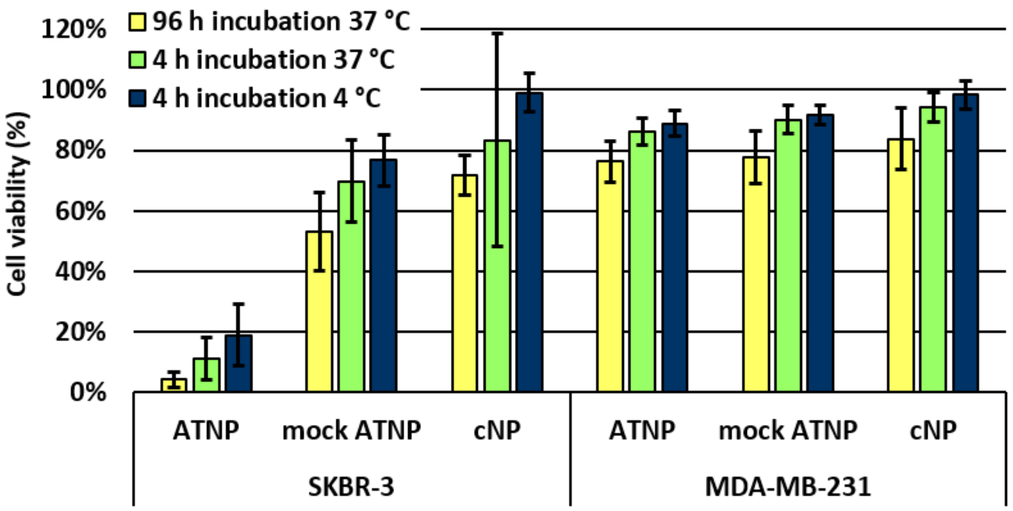

Figure S8: Both HER2$^+$ SKBR-3 and the HER2$^-$ MDA-MB-231 cells were incubated with 45 nM (particle concentration) of either: the ATNP (T-ssDNA/micelle Antibody-Toxic Nanoparticle complex), the mock ATNP (R-ssDNA/micelle complex) or the cNP (cationic micelle). Either the cells were incubated with the aforementioned compounds at 37 °C for 96 h (yellow bars); or they were incubated 4 h at 37 °C with the compounds, then the medium was replaced with fresh culture medium (DMEM, 10% FBS), and incubated at 37 °C for the remaining 92 h (green bars); or they were incubated 4 h at 4 °C with the compounds, then the medium was replaced with fresh culture medium (DMEM, 10% FBS), and incubated at 37 °C for the remaining 92 h (blue bars). The viability was then measured using the MTT assay. The ATNP complex were prepared at a molar ratio of 0.4 AOC/cNP. The viability is expressed as a percentage, relative to vehicle-treated (i.e. 20 µL of pure HBG buffer), and the error bars represent the standard deviation calculated from at least 3 independent assays.

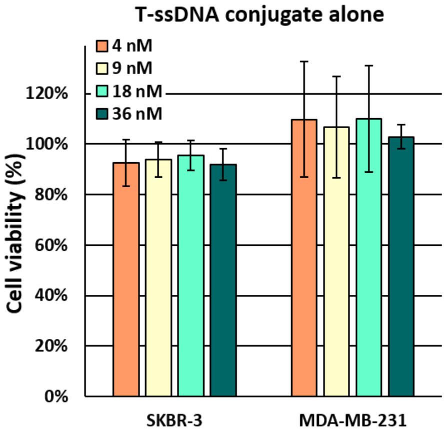

Figure S9: The cellular viability of both the HER2$^+$ SKBR-3 and the HER2$^-$ MDA-MB-231 cell lines after 96 h of continuous incubation at 37 °C in culture medium, in the presence of trastuzumab-ssDNA conjugate (T-ssDNA) at concentrations of 4, 9, 18 and 36 nM (corresponding to the concentrations of T-ssDNA involved in ATNP complexes at concentrations of 11, 23, 45, and 91 nM, respectively), was measured using the MTT assay. The viability is expressed as a percentage, relative to vehicle-treated (i.e. 20 µL of pure HBG buffer), and the error bars represent the standard deviation calculated from at least 3 independent assays.

In vivo experiments

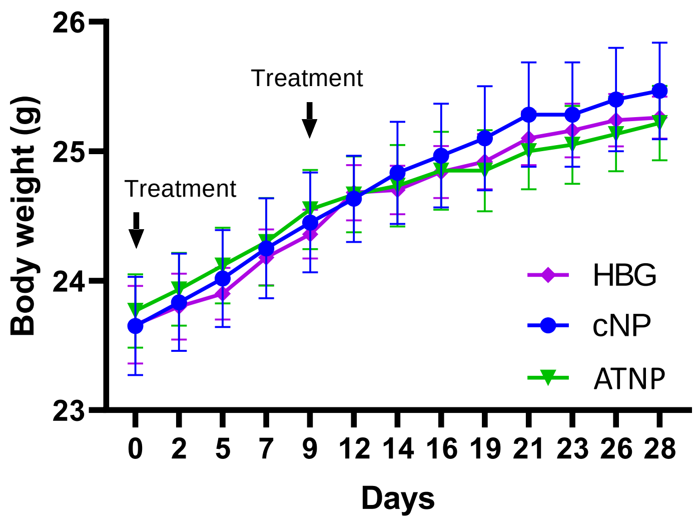

Figure S1: No toxicity was highlight by body weight monitoring of the three group of animals treated two times with vehicle HBG as control group, or cationic nanoparticles (cNP, 1.8 pmol/kg) and Antibody toxic Nanoparticle conjugate (ATNP, 1.8 pmol/kg)

REFERENCES

(1) Beck, A.; Goetsch, L.; Dumontet, C.; Corvaïa, N. Strategies and Challenges for the next Generation of Antibody-Drug Conjugates. *Nat. Rev. Drug Discov.* **2017**, *16* (5), 315–337. https://doi.org/10.1038/nrd.2016.268.

(2) Weldon, J. E.; Pastan, I. A Guide to Taming a Toxin: Recombinant Immunotoxins Constructed from Pseudomonas Exotoxin A for the Treatment of Cancer. *FEBS J.* **2011**, *278* (23), 4683. https://doi.org/10.1111/j.1742-4658.2011.08182.x.

(3) Lutz, R. J. CHAPTER 22:The Future of Antibody–Drug Conjugate (ADC) Payloads. In *Cytotoxic Payloads for Antibody–Drug Conjugates*; 2019; pp 461–471. https://doi.org/10.1039/9781788012898-00461.

(4) Khongorzul, P.; Ling, C. J.; Khan, F. U.; Ihsan, A. U.; Zhang, J. Antibody–Drug Conjugates: A Comprehensive Review. *Mol. Cancer Res.* **2020**, *18* (1), 3–19.

(5) Gingrich, J.; Ph.D. *How Next Generation ADCs Expand Beyond Cytotoxic Payloads*. ADC Review. https://www.adcreview.com/articles/how-the-next-generation-antibody-drug-conjugates-expands-beyond-cytotoxic-payloads-for-cancer-therapy/ (accessed 2021-07-08).

(6) Dovgan, I.; Koniev, O.; Kolodych, S.; Wagner, A. Antibody–Oligonucleotide Conjugates as Therapeutic, Imaging, and Detection Agents. *Bioconjug. Chem.* **2019**, *30* (10), 2483–2501. https://doi.org/10.1021/acs.bioconjchem.9b00306.

(7) Mi, P.; Cabral, H.; Kataoka, K. Ligand-Installed Nanocarriers toward Precision Therapy. *Adv. Mater.* **2020**, *32* (13), 1902604. https://doi.org/10.1002/adma.201902604.

(8) Kumar, R.; Santa Chalarca, C. F.; Bockman, M. R.; Bruggen, C. V.; Grimme, C. J.; Dalal, R. J.; Hanson, M. G.; Hexum, J. K.; Reineke, T. M. Polymeric Delivery of Therapeutic Nucleic Acids. *Chem. Rev.* **2021**. https://doi.org/10.1021/acs.chemrev.0c00997.

(9) Roberts, T. C.; Langer, R.; Wood, M. J. A. Advances in Oligonucleotide Drug Delivery. *Nat. Rev. Drug Discov.* **2020**, *19* (10), 673–694. https://doi.org/10.1038/s41573-020-0075-7.

(10) Chernikov, I. V.; Vlassov, V. V.; Chernolovskaya, E. L. Current Development of SiRNA Bioconjugates: From Research to the Clinic. *Front. Pharmacol.* **2019**, *10*. https://doi.org/10.3389/fphar.2019.00444.

(11) Zhou, Z.; Liu, X.; Zhu, D.; Wang, Y.; Zhang, Z.; Zhou, X.; Qiu, N.; Chen, X.; Shen, Y. Nonviral Cancer Gene Therapy: Delivery Cascade and Vector Nanoproperty Integration. *Adv. Drug Deliv. Rev.* **2017**, *115*, 115–154. https://doi.org/10.1016/j.addr.2017.07.021.

(12) Gómez-Aguado, I.; Rodríguez-Castejón, J.; Vicente-Pascual, M.; Rodríguez-Gascón, A.; Solinís, M. Á.; del Pozo-Rodríguez, A. Nanomedicines to Deliver MRNA: State of the Art and Future Perspectives. *Nanomaterials* **2020**, *10* (2), 364. https://doi.org/10.3390/nano10020364.

(13) Ben Djemaa, S.; Munnier, E.; Chourpa, I.; Allard-Vannier, E.; David, S. Versatile Electrostatically Assembled Polymeric SiRNA Nanovectors: Can They Overcome the Limits of SiRNA Tumor Delivery? *Int. J. Pharm.* **2019**, *567*, 118432. https://doi.org/10.1016/j.ijpharm.2019.06.023.

(14) Chen, J.; Wang, K.; Wu, J.; Tian, H.; Chen, X. Polycations for Gene Delivery: Dilemmas and Solutions. *Bioconjug. Chem.* **2019**, *30* (2), 338–349. https://doi.org/10.1021/acs.bioconjchem.8b00688.
(15) Jeon, T.; Luther, D. C.; Goswami, R.; Bell, C.; Nagaraj, H.; Cicek, Y. A.; Huang, R.; Mas-Rosario, J. A.; Elia, J. L.; Im, J.; Lee, Y.-W.; Liu, Y.; Scaletti, F.; Farkas, M. E.; Mager, J.; Rotello, V. M. Engineered Polymer–SiRNA Polyplexes Provide Effective Treatment of Lung Inflammation. *ACS Nano* **2023**, *17* (5), 4315–4326. https://doi.org/10.1021/acsnano.2c08690.
(16) Moghimi, S. M.; Symonds, P.; Murray, J. C.; Hunter, A. C.; Debska, G.; Szewczyk, A. A Two-Stage Poly(Ethylenimine)-Mediated Cytotoxicity: Implications for Gene Transfer/Therapy. *Mol. Ther.* **2005**, *11* (6), 990–995. https://doi.org/10.1016/j.ymthe.2005.02.010.
(17) Choi, Y. J.; Kang, S. J.; Kim, Y. J.; Lim, Y.; Chung, H. W. Comparative Studies on the Genotoxicity and Cytotoxicity of Polymeric Gene Carriers Polyethylenimine (PEI) and Polyamidoamine (PAMAM) Dendrimer in Jurkat T-Cells. *Drug Chem. Toxicol.* **2010**, *33* (4), 357–366. https://doi.org/10.3109/01480540903493507.
(18) Hunter, A. C. Molecular Hurdles in Polyfectin Design and Mechanistic Background to Polycation Induced Cytotoxicity. *Adv. Drug Deliv. Rev.* **2006**, *58* (14), 1523–1531. https://doi.org/10.1016/j.addr.2006.09.008.
(19) Roursgaard, M.; Knudsen, K. B.; Northeved, H.; Persson, M.; Christensen, T.; Kumar, P. E. K.; Permin, A.; Andresen, T. L.; Gjetting, T.; Lykkesfeldt, J.; Vesterdal, L. K.; Loft, S.; Møller, P. In Vitro Toxicity of Cationic Micelles and Liposomes in Cultured Human Hepatocyte (HepG2) and Lung Epithelial (A549) Cell Lines. *Toxicol. Vitro Int. J. Publ. Assoc. BIBRA* **2016**, *36*, 164–171. https://doi.org/10.1016/j.tiv.2016.08.002.
(20) Monnery, B. D.; Wright, M.; Cavill, R.; Hoogenboom, R.; Shaunak, S.; Steinke, J. H. G.; Thanou, M. Cytotoxicity of Polycations: Relationship of Molecular Weight and the Hydrolytic Theory of the Mechanism of Toxicity. *Int. J. Pharm.* **2017**, *521* (1), 249–258. https://doi.org/10.1016/j.ijpharm.2017.02.048.
(21) Blanco, E.; Shen, H.; Ferrari, M. Principles of Nanoparticle Design for Overcoming Biological Barriers to Drug Delivery. *Nat. Biotechnol.* **2015**, *33* (9), 941–951. https://doi.org/10.1038/nbt.3330.
(22) Lochbaum, C. A.; Chew, A. K.; Zhang, X.; Rotello, V.; Van Lehn, R. C.; Pedersen, J. A. Lipophilicity of Cationic Ligands Promotes Irreversible Adsorption of Nanoparticles to Lipid Bilayers. *ACS Nano* **2021**, *15* (4), 6562–6572. https://doi.org/10.1021/acsnano.0c09732.
(23) Fröhlich, E. The Role of Surface Charge in Cellular Uptake and Cytotoxicity of Medical Nanoparticles. *Int. J. Nanomedicine* **2012**, *7*, 5577–5591. https://doi.org/10.2147/IJN.S36111.
(24) Dufès, C.; Keith, W. N.; Bilsland, A.; Proutski, I.; Uchegbu, I. F.; Schätzlein, A. G. Synthetic Anticancer Gene Medicine Exploits Intrinsic Antitumor Activity of Cationic Vector to Cure Established Tumors. *Cancer Res.* **2005**, *65* (18), 8079–8084. https://doi.org/10.1158/0008-5472.CAN-04-4402.
(25) Kim, C.; Agasti, S. S.; Zhu, Z.; Isaacs, L.; Rotello, V. M. Recognition-Mediated Activation of Therapeutic Gold Nanoparticles inside Living Cells. *Nat. Chem.* **2010**, *2* (11), 962–966. https://doi.org/10.1038/nchem.858.
(26) Dai, Q.; Bertleff-Zieschang, N.; Braunger, J. A.; Björnmalm, M.; Cortez-Jugo, C.; Caruso, F. Particle Targeting in Complex Biological Media. *Adv. Healthc. Mater.* **2018**, *7* (1), 1700575. https://doi.org/10.1002/adhm.201700575.
(27) Carter, T.; Mulholland, P.; Chester, K. Antibody-Targeted Nanoparticles for Cancer Treatment. *Immunotherapy* **2016**, *8* (8), 941–958. https://doi.org/10.2217/imt.16.11.
(28) Wen, Y.; Bai, H.; Zhu, J.; Song, X.; Tang, G.; Li, J. A Supramolecular Platform for Controlling and Optimizing Molecular Architectures of SiRNA Targeted Delivery Vehicles. *Sci. Adv.* **2020**, *6* (31), eabc2148. https://doi.org/10.1126/sciadv.abc2148.
(29) Kim, S. H.; Jeong, J. H.; Chun, K. W.; Park, T. G. Target-Specific Cellular Uptake of PLGA Nanoparticles Coated with Poly(l-Lysine)−Poly(Ethylene Glycol)−Folate Conjugate. *Langmuir* **2005**, *21* (19), 8852–8857. https://doi.org/10.1021/la0502084.
(30) Green, J. J.; Chiu, E.; Leshchiner, E. S.; Shi, J.; Langer, R.; Anderson, D. G. Electrostatic Ligand Coatings of Nanoparticles Enable Ligand-Specific Gene Delivery to Human Primary Cells. *Nano Lett.* **2007**, *7* (4), 874–879. https://doi.org/10.1021/nl062395b.
(31) Eltoukhy, A. A.; Chen, D.; Veiseh, O.; Pelet, J. M.; Yin, H.; Dong, Y.; Anderson, D. G. Nucleic Acid-Mediated Intracellular Protein Delivery by Lipid-like Nanoparticles. *Biomaterials* **2014**, *35* (24), 6454–6461. https://doi.org/10.1016/j.biomaterials.2014.04.014.

(32) Theodorou, I.; Anilkumar, P.; Lelandais, B.; Clarisse, D.; Doerflinger, A.; Gravel, E.; Ducongé, F.; Doris, E. Stable and Compact Zwitterionic Polydiacetylene Micelles with Tumor-Targeting Properties. *Chem. Commun.* **2015**, *51* (80), 14937–14940. https://doi.org/10.1039/C5CC05333A.
(33) Neuberg, P.; Perino, A.; Morin-Picardat, E.; Anton, N.; Darwich, Z.; Weltin, D.; Mely, Y.; Klymchenko, A. S.; Remy, J.-S.; Wagner, A. Photopolymerized Micelles of Diacetylene Amphiphile: Physical Characterization and Cell Delivery Properties. *Chem. Commun.* **2015**, *51* (58), 11595–11598. https://doi.org/10.1039/C5CC03820K.
(34) Ripoll, M.; Neuberg, P.; Kichler, A.; Tounsi, N.; Wagner, A.; Remy, J.-S. PH-Responsive Nanometric Polydiacetylenic Micelles Allow for Efficient Intracellular SiRNA Delivery. *ACS Appl. Mater. Interfaces* **2016**, *8* (45), 30665–30670. https://doi.org/10.1021/acsami.6b09365.
(35) Neuberg, P.; Wagner, A.; Remy, J.-S.; Kichler, A. Design and Evaluation of Ionizable Peptide Amphiphiles for SiRNA Delivery. *Int. J. Pharm.* **2019**, *566*, 141–148. https://doi.org/10.1016/j.ijpharm.2019.05.052.
(36) Midoux, P.; Pichon, C.; Yaouanc, J.-J.; Jaffrès, P.-A. Chemical Vectors for Gene Delivery: A Current Review on Polymers, Peptides and Lipids Containing Histidine or Imidazole as Nucleic Acids Carriers. *Br. J. Pharmacol.* **2009**, *157* (2), 166–178. https://doi.org/10.1111/j.1476-5381.2009.00288.x.
(37) Wen, Y.; Guo, Z.; Du, Z.; Fang, R.; Wu, H.; Zeng, X.; Wang, C.; Feng, M.; Pan, S. Serum Tolerance and Endosomal Escape Capacity of Histidine-Modified PDNA-Loaded Complexes Based on Polyamidoamine Dendrimer Derivatives. *Biomaterials* **2012**, *33* (32), 8111–8121. https://doi.org/10.1016/j.biomaterials.2012.07.032.
(38) Wang, F.; Hu, K.; Cheng, Y. Structure–Activity Relationship of Dendrimers Engineered with Twenty Common Amino Acids in Gene Delivery. *Acta Biomater.* **2016**, *29*, 94–102. https://doi.org/10.1016/j.actbio.2015.10.034.
(39) Ewe, A.; Przybylski, S.; Burkhardt, J.; Janke, A.; Appelhans, D.; Aigner, A. A Novel Tyrosine-Modified Low Molecular Weight Polyethylenimine (P10Y) for Efficient SiRNA Delivery in Vitro and in Vivo. *J. Controlled Release* **2016**, *230*, 13–25. https://doi.org/10.1016/j.jconrel.2016.03.034.
(40) Leyton, J. V. Improving Receptor-Mediated Intracellular Access and Accumulation of Antibody Therapeutics—The Tale of HER2. *Antibodies* **2020**, *9* (3), 32. https://doi.org/10.3390/antib9030032.
(41) Du, J.; Wang, H.; Zhong, C.; Peng, B.; Zhang, M.; Li, B.; Huo, S.; Guo, Y.; Ding, J. Structural Basis for Recognition of CD20 by Therapeutic Antibody Rituximab. *J. Biol. Chem.* **2007**, *282* (20), 15073–15080. https://doi.org/10.1074/jbc.M701654200.
(42) Ripoll, M.; Neuberg, P.; Wagner, A.; Remy, J.-S. Amphiphilic Monomers Based Nanovectors and Their Use for Sirna Delivery. WO/2017/167929, October 5, 2017. https://patentscope.wipo.int/search/en/detail.jsf?docId=WO2017167929 (accessed 2021-07-09).
(43) Dovgan, I.; Ursuegui, S.; Erb, S.; Michel, C.; Kolodych, S.; Cianférani, S.; Wagner, A. Acyl Fluorides: Fast, Efficient, and Versatile Lysine-Based Protein Conjugation via Plug-and-Play Strategy. *Bioconjug. Chem.* **2017**, *28* (5), 1452–1457. https://doi.org/10.1021/acs.bioconjchem.7b00141.
(44) Lehot, V.; Kuhn, I.; Nothisen, M.; Erb, S.; Kolodych, S.; Cianférani, S.; Chaubet, G.; Wagner, A. Non-Specific Interactions of Antibody-Oligonucleotide Conjugates with Living Cells. *Sci. Rep.* **2021**, *11* (1), 5881. https://doi.org/10.1038/s41598-021-85352-w.
(45) Zhao, Y. H.; Abraham, M. H.; Zissimos, A. M. Fast Calculation of van Der Waals Volume as a Sum of Atomic and Bond Contributions and Its Application to Drug Compounds. *J. Org. Chem.* **2003**, *68* (19), 7368–7373. https://doi.org/10.1021/jo034808o.
(46) Ripoll, M.; Neuberg, P.; Remy, J.-S.; Kichler, A. Cationic Photopolymerized Polydiacetylenic (PDA) Micelles for SiRNA Delivery. *Methods Mol. Biol. Clifton NJ* **2019**, *1943*, 101–122. https://doi.org/10.1007/978-1-4939-9092-4_7.
(47) Ripoll, M. Synthèse de Nano-Vecteurs Dérivés Des Polydiacétylènes Pour La Co-Délivrance d'un ARN Interférent et d'un Anticancéreux. These de doctorat, Strasbourg, 2017. http://www.theses.fr/2017STRAF076 (accessed 2021-07-21).
(48) Thielens, N. M.; Belime, A.; Gravel, E.; Ancelet, S.; Caneiro, C.; Doris, E.; Ling, W. L. Impact of the Surface Charge of Polydiacetylene Micelles on Their Interaction with Human Innate Immune Protein C1q and the Complement System. *Int. J. Pharm.* **2018**, *536* (1), 434–439. https://doi.org/10.1016/j.ijpharm.2017.11.072.
(49) Wei, Y.; Quan, L.; Zhou, C.; Zhan, Q. Factors Relating to the Biodistribution & Clearance of Nanoparticles & Their Effects on in Vivo Application. *Nanomed.* **2018**, *13* (12), 1495–1512. https://doi.org/10.2217/nnm-2018-0040.

(50) Mitchell, M. J.; Billingsley, M. M.; Haley, R. M.; Wechsler, M. E.; Peppas, N. A.; Langer, R. Engineering Precision Nanoparticles for Drug Delivery. *Nat. Rev. Drug Discov.* **2021**, *20* (2), 101–124. https://doi.org/10.1038/s41573-020-0090-8.
(51) Salvati, A.; Pitek, A. S.; Monopoli, M. P.; Prapainop, K.; Bombelli, F. B.; Hristov, D. R.; Kelly, P. M.; Åberg, C.; Mahon, E.; Dawson, K. A. Transferrin-Functionalized Nanoparticles Lose Their Targeting Capabilities When a Biomolecule Corona Adsorbs on the Surface. *Nat. Nanotechnol.* **2013**, *8* (2), 137–143. https://doi.org/10.1038/nnano.2012.237.
(52) Bros, M.; Nuhn, L.; Simon, J.; Moll, L.; Mailänder, V.; Landfester, K.; Grabbe, S. The Protein Corona as a Confounding Variable of Nanoparticle-Mediated Targeted Vaccine Delivery. *Front. Immunol.* **2018**, *9*. https://doi.org/10.3389/fimmu.2018.01760.
(53) Baran, A.; Fırat Baran, M.; Keskin, C.; Hatipoğlu, A.; Yavuz, Ö.; İrtegün Kandemir, S.; Adican, M. T.; Khalilov, R.; Mammadova, A.; Ahmadian, E.; Rosić, G.; Selakovic, D.; Eftekhari, A. Investigation of Antimicrobial and Cytotoxic Properties and Specification of Silver Nanoparticles (AgNPs) Derived From Cicer Arietinum L. Green Leaf Extract. *Front. Bioeng. Biotechnol.* **2022**, *10*.
(54) Sellami, H.; Khan, S. A.; Ahmad, I.; Alarfaj, A. A.; Hirad, A. H.; Al-Sabri, A. E. Green Synthesis of Silver Nanoparticles Using Olea Europaea Leaf Extract for Their Enhanced Antibacterial, Antioxidant, Cytotoxic and Biocompatibility Applications. *Int. J. Mol. Sci.* **2021**, *22* (22), 12562. https://doi.org/10.3390/ijms222212562.
